# When a family physician grieves: Facing loss as a daughter who is a doctor

**DOI:** 10.51866/mol.1178

**Published:** 2026-07-29

**Authors:** Ilham Ameera Ismail

**Affiliations:** 1 Department of Community and Ambulatory Services, Hospital Al-Sultan Abdullah, Universiti Teknologi MARA, Cawangan Selangor, Kampus Puncak Alam, Malaysia.; 2 Department of Primary Care Medicine, Faculty of Medicine, Universiti Teknologi MARA, Cawangan Selangor, Kampus Sungai Buloh, Malaysia.

**Keywords:** Bereavement, Coping, Family medicine specialist

In the consultation room, I have often held the space for others’ sorrow. I have delivered bad news with a practised, steady hand, offering standard management plans for the bereaved. I knew the steps of breaking bad news, the stages of grief and what to expect. However, when my father passed away, the clinician in me was suddenly and violently replaced by the grieving daughter. I found that having a medical degree and being a primary care specialist offer no immunity to the raw, jagged edges of personal loss.

Physicians could experience disenfranchised grief, a loss not openly acknowledged or socially supported due to the perceived sanctioning of emotions in professional settings.^1^ They are conditioned towards professional stoicism, expected to return to the clinic, to work and to remain the pillar of strength for the community, even when their internal landscape is in ruins.

In my case, my grieving period was further complicated by my proximity to the community. As a primary care physician, I often see my own father in my long-term older adult patients, a dynamic that can lead to intense internal conflict when professional obligations overlap with personal mourning. I am forced to balance the public face of a doctor with the private shadow of a grieving family member.

Looking back, during my father’s final days, I found myself oscillating between being his daughter and being a doctor. I was often the reference person for the family in explaining his condition medically. I knew that his condition was worsening and we were losing him, but it was all explained from a medical perspective. This is a defence mechanism known as intellectualisation.^2^ Intellectualisation allows physicians to distance themselves from unacceptable or threatening feelings by keeping a cognitive focus on the medical problem rather than the emotional reality.^2^ Yet, focusing solely on procedural details without working through the grief only adds to their psychological burden.^3^ My experience was further complicated by ‘rescue fantasies’^4^ and the recurring internal inquiry of whether I had fulfilled my dual obligations as a ‘doctor–daughter’ while carefully avoiding any breach of medical ethics.

Physicians also grieve. I now realise that it is acceptable to take the time to grieve properly and that the way physicians grieve might be influenced by the profession.^5^ As it is a mixture of family and professional bereavement, coping strategies used by healthcare professionals in response to bereavement can be uncommon, unique and multidimensional.^3^

It is also important to understand that although physicians are supposed to be experts on what to do, they could still need help from others. They need to understand their grief as physicians and how to convert anguish to an opportunity for personal growth and better patient care.^5^ My experience provided me with a deeper understanding that the best management plan for grief is not a prescription but the grace to simply be human.

**Figure 1:**
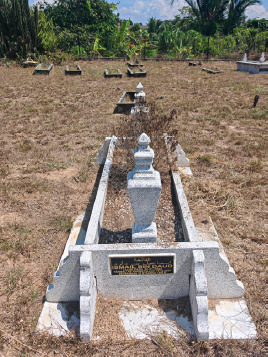
My father’s grave.

Al-Fatihah to Haji Ismail bin Daud, a beloved father who is missed daily. His grave is shown in Figure 1.
